# Glaucoma Ex-PRESS Implantation Surgery: Is There a Difference in Efficacy and Safety between Inpatient and Outpatient Management?

**DOI:** 10.31662/jmaj.2023-0216

**Published:** 2024-06-03

**Authors:** Yurika Aoyama, Rei Sakata, Takashi Fujishiro, Makoto Aihara

**Affiliations:** 1 Department of Ophthalmology, Graduate of Medicine and Faculty of Medicine, The University of Tokyo, Tokyo, Japan; 2 Yotsuya Shirato Eye Clinic, Tokyo, Japan

**Keywords:** glaucoma, filtration surgery, inpatient, outpatient, intraocular pressure

## Introduction

Glaucoma is the leading cause of visual impairment in Japan, and blindness prevention is a pressing issue ^(1)^. At present, evidence-based treatment for glaucoma is limited to intraocular pressure (IOP)-lowering therapies ^(2)^, and the choice of treatment among eye drops, laser procedures, and surgery is determined based on individual cases ^(3)^. The most effective surgical procedure is filtration surgery; however, this technique is commonly performed as an inpatient procedure in Japan to reduce early postoperative burden (frequency of visits) on patients ^(4)^. However, concerns regarding the potential risk of delirium and increased frailty associated with prolonged hospital stays, particularly among the aging population, have been raised ^(5)^. The advantage of inpatient management is that daily examinations can be conducted, but there is a tendency for an extended length of stay, which contributes to an increase in medical expenses. To date, no comparative studies have evaluated the differences of inpatient versus outpatient filtration surgeries in terms of IOP control and safety. Therefore, we analyzed the short-term IOP reduction rate and safety of patients undergoing filtration surgery performed by a single surgeon during the critical postoperative period, specifically the first 3 months after surgery.

## Material and Methods

**Figure 1.**
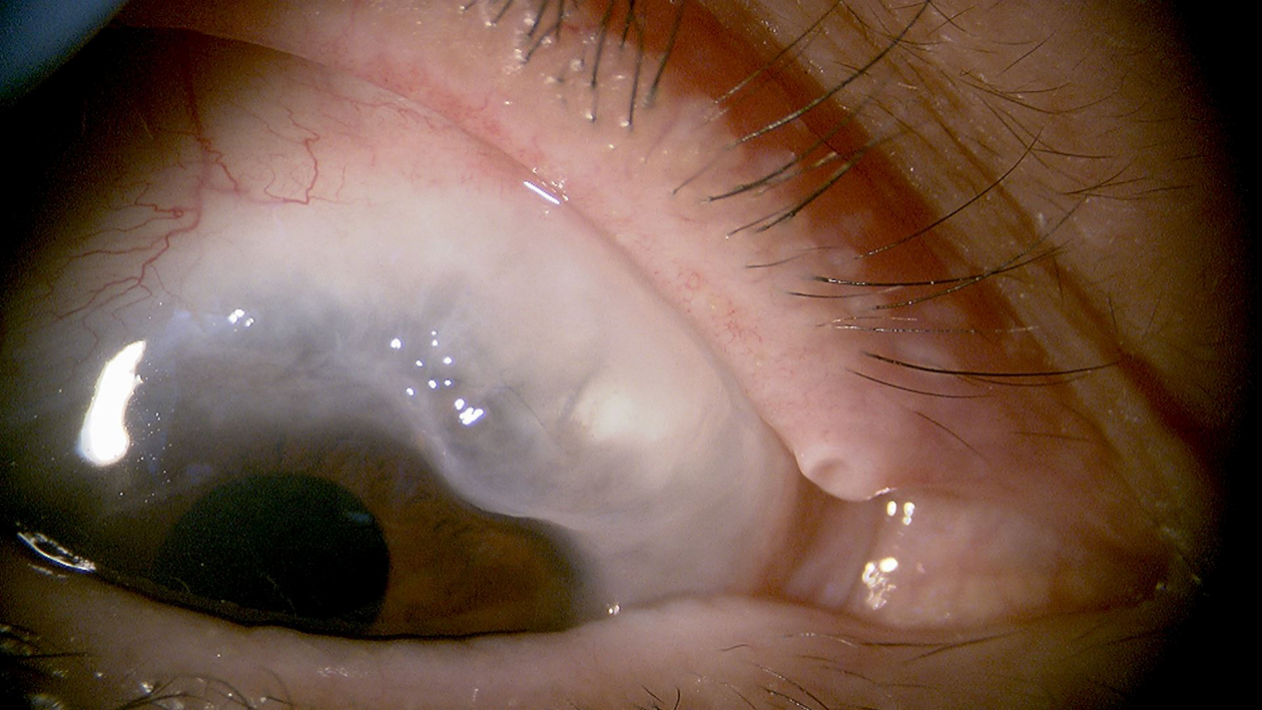
Filtration bleb morphology following filtration surgery This photograph (right eye) was taken within a certain period after filtration surgery, during which the patients’ condition was stable. Filtration surgery is a procedure that creates a new pathway for aqueous humor to flow under the conjunctiva. When a filtration bleb is created beneath the conjunctiva, aqueous humor flows into that area, resulting in the formation of a white bleb.

This retrospective study included Japanese patients with glaucoma who underwent filtration surgery (Ex-PRESS, Alcon, Fort Worth, TX, USA) between March 2018 and March 2021. The choice between inpatient and outpatient postoperative management depended on the patient’s preferences. Consecutive patients with a 3-month follow-up were included, whereas those with angle-closure glaucoma and a history of glaucoma surgery were excluded. The study was conducted in accordance with the principles of the Declaration of Helsinki. The study protocol was approved by the ethics committee (ID: 2217-8). Written informed consent was obtained from all patients for study participation. The Ex-PRESS procedure was performed by a single surgeon (MA) as previously described ^(6)^. Briefly, the procedure was performed using a fornix-based approach, and subconjunctival administration of 0.05% mitomycin C for 1.5 min was initiated (Figure 1). Postoperative treatment included four applications of 0.1% betamethasone and moxifloxacin ophthalmic solution. Combined cataract surgery involved diclofenac sodium ophthalmic solution. Laser suture lysis or bleb needling was performed to increase the aqueous humor into the subconjunctiva at the surgeon’s discretion.

Inpatient management lasted approximately 1 week, and patients were required to undergo two ophthalmic examinations per day. Conversely, outpatient management included examinations on the following day and then on the 4^th^ and 7^th^ days. In both groups, subsequent appointments were made at intervals of 1-2 weeks from the 7^th^ day onward at an outpatient clinic, and from the 1st month, appointments were made at intervals of 4-6 weeks. However, in cases where needling or additional suturing was performed, a follow-up visit was scheduled 1 week later.

We compared the IOP reduction rate at 3 months and the total number of ophthalmic examinations between the inpatient and outpatient management groups for 3 months. Fisher’s exact probability test was employed to compare the proportion of cases achieving an IOP reduction rate of 30% (without any medications) at 3 months between the inpatient and outpatient management. Logistic regression analysis was conducted to evaluate the impact of treatment modality (inpatient or outpatient) and the total number of ophthalmic examinations on the IOP reduction rate ≥30%. The rate of 30% was determined based on findings conducted in Japan ^(7)^. The IOP reduction rate (%) was calculated using the following formula: ((postoperative IOP) - (preoperative IOP)) × 100 / (preoperative IOP). Statistical analysis was conducted using JMP^Ⓡ^ Pro (version 17.0). *P* < 0.05 was considered to indicate statistical significance.

## Results

A total of 45 consecutive patients were enrolled (20 eyes of inpatient management group [IMG] and 25 eyes of outpatient management group [OMG]). The total number of ophthalmic examinations from postoperative period to the third month was 16.0 ± 3.8 times in IMG and 5.6 ± 0.6 times in OMG (*P* < 0.001) (Table 1).

**Table 1.** Clinical Characteristics.

	Inpatient management group	Outpatient management group	*P*-value
Age (years)	62.8 ± 10.0	64.2 ± 8.3	0.61
Sex (M/F)	9/11	13/12	0.77
Diagnosis (number)			0.55
POAG	10	13
NTG	7	11
PEG	2	1
Secondary glaucoma	1	0
Preoperative IOP (mmHg)	17.8 ± 3.5	18.0 ± 3.7	0.83
Eye drops (number)	3.5 ± 1.2	3.7 ± 0.9	0.58
Refraction (Diopters)	−5.7 ± 4.7	−3.3 ± 2.9	0.056
Number of ophthalmic examinations* (times)	16.0	5.6	<.001
Needling* (number)	9	3	0.019
Laser suture lysis* (mean number)	0.95	0.76	0.66

M, male; F, female; POAG, primary open-angle glaucoma; NTG, normal-tension glaucoma; PEG, pseudoexfoliation glaucoma; IOP, intraocular pressure *during follow-up

The postoperative IOP values for the IMG and OMG were 7.4 and 6.9 mmHg at 1 week, 13.0 and 8.6 mmHg at 1 month, and 15.0 and 9.5 mmHg at 3 months, respectively. For the IMG and OMG, the rates of achieving a mean IOP of less than 12 mmHg at 3 months were 60% and 96% whereas those for IOP of less than 14 mmHg were 75% and 100%, respectively.

At the 3-month postoperative follow-up, 60% of the IMG and 68% of the OMG achieved an IOP reduction ≥30% (Fisher’s exact test, *P* = 0.76). The odds ratio for achieving an IOP reduction rate ≥30% for OMG was 0.71 (95% confidence interval [CI]: 0.21-2.41; *P* = 0.58) (with IMG as the reference); similarly, the odds ratio for achieving an IOP reduction rate ≥30% for the number of ophthalmic examinations was 1.01 (95% CI: 0.90-1.13; *P* = 0.88) (Table 2). Postoperative complications included an additional suture in one eye for IMG and shallow anterior chamber and choroidal detachment in three eyes for OMG. All of them were nonserious adverse events that did not affect visual function.

**Table 2.** Results of Logistic Regression Analysis of IOP Reduction Rate over 30%.

Covariates	Odds ratio (95% CI)	*P*-value
Age	1.03 (0.96-1.11)	0.36
Sex (reference as male)	2.05 (0.59-7.15)	0.26
Refraction	1.15 (0.95-1.38)	0.13
Preoperative lens status (reference as pseudophakia)	0.96 (0.28-3.27)	0.94
Preoperative IOP	0.69 (0.53-0.91)	0.002
Preoperative medication score	1.04 (0.57-1.90)	0.90
Treatment type (reference as inpatient management)	0.71 (0.21-2.41)	0.58
Number of ophthalmic examinations	1.01 (0.90-1.13)	0.88

IOP, intraocular pressure; CI, confidence interval

## Discussion

Cataract surgery in ophthalmology is usually performed as outpatient management rather than inpatient management. However, glaucoma filtration surgery, which requires postoperative close IOP management, is commonly performed under inpatient care in Japan. In this study, no significant difference was observed in the IOP reduction rate (>30%) at the 3-month postoperative mark between inpatient and outpatient management. The results also indicated safety within acceptable limits.

Although inpatient management involved more frequent ophthalmic examinations, this was because in the inpatient management, residents were also involved in patient examinations along with the attending physician. During each examination, in addition to measuring IOP, the eyeball was tilted downward to observe the filtration bleb, which can inadvertently apply excessive force to the conjunctival suture site and exerted pressure on the filtration bleb with the fingers. Therefore, the cumulative stress on the surgical site was undoubtedly higher in IMG than in OMG. In the IMG, there were more instances of needling procedures, leading to a stronger induction of inflammation ^(8)^. Consequently, some cases (five eyes) experienced worsened IOP management (data not shown). Despite these potential drawbacks, no significant differences were observed in the IOP reduction rate between the IMG and OMG. Further investigation is warranted to understand the relationship between the frequency of examinations and IOP management. Inpatient management is a necessary treatment modality when outpatient visit is difficult due to visual or physical challenges. However, considering the limited human resources on the hospital ward, outpatient management can also be considered.

This study has several limitations. First, the retrospective nature of the study may introduce selection bias among patients, making it difficult to interpret the results in a general context. However, in this study, a single surgeon (35 years of experience) performed all surgeries, and surgical expertise was standardized, eliminating the need to consider confounding factors such as differences in surgical techniques. Second, this study was exploratory rather than confirmatory. It focused on patients undergoing the same surgery within a defined period and explored IOP management between the two groups without explicitly stating hypotheses. Consequently, the sample size was not adequately determined. To address this limitation, prospective randomized allocation studies are warranted. Third, glaucoma itself is a chronic progressive disease, and its ultimate goal is to maintain visual function, necessitating an examination from a long-term perspective, not only focusing on short-term outcomes. However, the 3-month postoperative period covered in this study is crucial for postoperative IOP management in filtration surgery ^(9)^, and it is also the period with the highest complication rate. It is essential to navigate through this period without major troubles as it is crucial for long-term IOP management. Therefore, focusing on the first several months postoperatively was deemed important. Lastly, as this study focused on Ex-PRESS implantation, its findings cannot be extrapolated to other types of glaucoma surgery such as trabeculectomy and valved tube surgery.

In conclusion, outpatient management in filtration surgery could not only offer patient convenience but also serve as a foundation for controlling the increased costs associated with inpatient care.

## Article Information

### Conflicts of Interest

None

### Author Contributions

Study conception and design: MA, Acquisition of data: YA, RS, TF, MA, Data interpretation: YA, RS, Drafting of the manuscript: YA, RS, Critical revision: MA

### Approval by Institutional Review Board (IRB)

ID: 2217-8, the University of Tokyo Hospital

### ORCID iD

Rei Sakata: https://orcid.org/0000-0003-1411-1476
